# The Medical Remembrancer, or Book of Emergencies; in Which Are Concisely Pointed out the Immediate Remedies to Be Adopted in the First Moments of Danger from Poisoning, Drowning, Apoplexy, Burns, and Other Accidents: With the Tests for the Principal Poisons, and Other Useful Information

**Published:** 1845-11

**Authors:** 


Art. XI.—*The Medical Remembrancer, or Book of Emergencies;* in which are concisely pointed out the immediate remedies to be adopted in the first moments of danger from Poisoning, Drowning, Apoplexy, Burns, and other accidents: with the Tests for the principal Poisons, and other Useful Information. By Edward B. L. Shaw, M.R.C.S. & L.A.S., one of the Surgeons to the Royal Humane Society, and Assistant Apothecary to St. Bartholomew's Hospital. Revised and improved by an American Physician. New York: Samuel S. & William Wood. 1845. 16mo. pp. 112.

The character and design of this little work are fully set forth in the words of the preface, which we subjoin:

"The number of works, large and small, on the subject of Toxicology, already in the hands of the Profession, renders it necessary to offer something in the way of apology for the appearance of so humble a publication as the present. To do this in a few words, it may be stated, that the want has been long felt among the junior members of the profession, of some practical guide in cases of emergency, which should be at once so portable as to admit of being conveniently carried in the pocket, and so concise as to present at a glance the most appropriate remedy for the case, unencumbered with other matters, which, however valuable in themselves, tend only, in the first moments of alarm and confusion, to distract the attention from the object of primary importance. With this view, the *diagnostic symptoms*, and the *chemical analysis* of the different poisons, have been omitted in the following pages; the former, because there occur very few cases in which any doubt exists as to the particular agent the symptoms have been occasioned by; the latter, because chiefly in requisition after death. The immediate steps to be pursued, then, is all that is here endeavored to be shown; and the time consumed in turning over a large volume in quest of them (supposing one to be at hand, which is frequently not the case), induced the author to compile the present Manual for the use of his own pupils. And when it is considered how often the responsible charge of a surgery or chemist's shop is committed to an inexperienced young man, who may, while thus employed, be called upon at a moment's notice to prescribe in a case of extreme danger, an attempt, though imperfect, like the present, to point out the proper remedy, and thus prevent perhaps a fatal error, may not be deemed altogether useless. Upon this plea alone, it is submitted to the profession and the public, in the words of Ovid—

> "Da veniam scriptis quorum non gloria nobis,
> Causa, sed utilitas officiumque fuit."

It begins with an alphabetical catalogue of the most *common poisons*, with the immediate treatment to be adopted in case of their being taken into the stomach. To this follows a chapter on some of the *accidents* of more common occurrence, requiring medical assistance, and the appropriate means to be adopted in each case. Among these accidents are *abrasion of the skin*, *apoplexy*, *asphyxia* from various causes, *bite of rabid animals*, *&c.*, arranged, as in the former chapter, alphabetically. This plan renders the work exceedingly convenient for reference. We quote the treatment for *Croup* as laid down by the American editor.

"If the child be vigorous and above one year, use a solution of tartar emetic, one grain to the ounce of water, in divided doses of a teaspoonful every quarter of an hour until free vomiting. If under one year, ipecac. in syrup (a teaspoonful repeated) or in powder is preferable. If the attack be violent, bloodletting from the arm or by cups or leeches will be proper. For an infant within one year, two leeches applied over the upper part of the sternum (or breast-bone), and one leech for each additional year. After free vomiting, a warm bath of 98° Fahrenheit for ten minutes. When removed from the bath, dry the body, place it in bed, and apply to the throat a cloth wrung out in first hot water, and afterward in spirits of turpentine. As the violent symptoms pass off, calomel with pulv. antim. every hour is recommended until the bowels are opened, and then followed by castor oil. In the latter stages the chief reliance is to be placed on the stimulating emetics. A plaster made with Scotch snuff and lard is sometimes advantageously applied to the chest. Tracheotomy is of little value."

The application of *cold* to the throat has lately been made in some cases of croup in this city with the most surprising benefit. In some instances, a towel dipped in cold water and wrapped around the neck, has acted like a charm. The remedy is deserving of trial, especially as it does not interfere with the use of other curative measures.

We quote next—the directions given for the removal of substances in the eye:

"A substance getting accidentally in the eye may either lie disengaged on its surface, or, having penetrated the external coat, may there remain fixed. In the former case, it is easily removed by a camel-hair pencil, or a piece of paper rolled into the size of a crow-quill, with the end softened in the mouth. It is very common for the substance to stick in the cornea, when, if it cannot be removed with a probe or fine forceps, the point of a lancet should be carefully passed under it so as to lift it out. If, however, the removal cannot be effected without considerable difficulty, it is better to leave it to be detached by ulceration, taking every precaution to keep off undue inflammation, by avoiding a strong light, fomenting with warm water, &c. To remove fine particles of gravel, lime, &c., the eye should be syringed with luke-warm water till free from them; enjoining the patient afterwards to abstain from worrying the eye, under the impression that the substance is still there, which the enlargement of some of the minute vessels makes him believe to be actually the case."

The third chapter treats of the *minor operations in Surgery*. The mode of applying electricity and galvanism, of cupping and bleeding, of making issues, as well as of performing the more formidable operations of laryngotomy, tracheotomy, and œsophagotomy, is described. The use of the stomach-pump, and vaccination are also topics embraced in this chapter. The compendious little treatise closes with an account of *chemical analysis*, and *the tests for the principal poisons.* It is not necessary for us to notice it further. It costs but a trifle, and will be found so useful a pocket companion, that every practitioner will procure a copy of it.

